# *De novo* OAB After ATOMS: An Underestimated Problem or a Rare Side Effect?

**DOI:** 10.3389/fsurg.2019.00072

**Published:** 2019-12-17

**Authors:** Sandra Schönburg, Wilhelm Bauer, Nasreldin Mohammed, Clemens Brössner, Paolo Fornara

**Affiliations:** ^1^Department of Urology and Kidney Transplantation, Martin Luther University, Halle (Saale), Germany; ^2^Department of Urology, Hospital Barmherzige Schwestern, Vienna, Austria

**Keywords:** male stress urinary incontinence, ATOMS, *de novo*, overactive bladder, management

## Abstract

**Background:** The urinary incontinence system ATOMS (A.M.I., Austria) generates suburethral compression through its sphincter cushion. To what extent the ATOMS may lead to overactive bladder (OAB) symptoms or which risk factors for these symptoms exist remain unknown to date. We report on our multicentre evaluation on the prevalence, status, and therapy of OAB after ATOMS.

**Methods:** Between 10/09 and 01/17, a total of 361 patients received an ATOMS device in Vienna and Halle. A prerequisite for surgery was persistent male stress urinary incontinence lasting at least 6 months after the primary intervention, as well as the failure of conservative treatment. Patients with a preoperative untreated anastomotic stricture or detrusor overactivity were excluded. In addition to continence and voiding parameters, patient's age, BMI, comorbidities, and pre-treatment strategies of the underlying disease and urinary incontinence were examined. If *de novo* OAB was present, urodynamics were used for further clarification. Statistical analysis was performed with GraphPad Prism 7® (GraphPad Software, Inc., La Jolla, USA), *p* < 0.05 considered significant.

**Results:** OAB presented 18 patients (4.9%). Regarding the degree of urinary incontinence as well as uroflowmetry, residual volume and comorbidities, patients with an OAB showed no differences compared to patients without an OAB (*p* < 0.05). Only previous radiotherapy or urinary incontinence surgery and urethral stricture interventions resulted in statistically significant differences based on the bivariate analysis (*p* = 0.030, *p* = 0.006, *p* = 0.007). The consecutive postoperative urodynamics revealed a sensory OAB in 17 patients and a low-compliance bladder in a patient with newly diagnosed insulin-dependent type II diabetes mellitus. OAB was treated with a standard dose of antimuscarinic drugs and for the low-compliance bladder with botulinum toxin type A.

**Conclusion:** OAB symptoms can occur after ATOMS implantation, but are rare and have no clear correlation to the incontinence device but rather are due to urinary incontinence-related underlying diseases and previous treatments.

## Background

The implantation of the adjustable transobturator urinary incontinence system (ATOMS, A.M.I., Agency for Medical Innovations, Feldkirch, Austria) to treat persistent male stress urinary incontinence (male SUI) is an established surgical procedure. Mid- and long-term follow-up data (1–5) as well as a meta-analysis of approximately 1,393 patients from 20 studies (13 retrospective and 7 prospective studies) have revealed a very good continence success rate, with 67% of patients achieving dryness and 23% of patients showing improvement (>50% improvement in incontinence compared to baseline) (6). Functionally, the ATOMS device generates suburethral compression by its sphincter cushion. To what extent symptoms of a “*de novo*” overactive bladder (OAB) can arise as a result, or consequently, which risk factors lead to an OAB after ATOMS implantation, is unknown to date. Therefore, the present study prospectively investigates the prevalence, status and therapy of OAB after ATOMS urinary incontinence surgery.

## Patients and Methods

In the present international multicentre prospective observational study (Vienna, Halle), we evaluated data for 361 patients with persistent male SUI who received an ATOMS between 10/09 and 01/17. Patient consent was obtained for the study. All patients were supervised during an individual incontinence consultation in an outpatient clinic. A prerequisite for surgery was persistent male SUI lasting at least 6 months after the primary intervention, as well as the failure of conservative treatment (e.g., pelvic floor exercises and biofeedback, electrotherapy, lifestyle modifications and anticholinergic medications). As part of the diagnostic and consultation process, we also evaluated the indications for other devices used to treat male SUI. For patients with only a slight urinary incontinence and a good residual sphincter function, a pubourethral sling may be an option, too. The case of complete incontinence without any residual sphincter function may be reserved to the artificial urinary sphincter. In the present study, however, only patients who received an ATOMS device were included. As previously described (4), the preoperative investigation contained a detailed medical history (especially regarding previous radiation and incontinence surgery), urinalysis, uroflowmetry, ultrasound with post-void residual volume, 3-day voiding protocol, 24-h pad count, and an urodynamic study to exclude detrusor overactivity as well as a diagnostic cystoscopy to exclude anastomotic stricture and to assess residual sphincter function. An untreated anastomotic stricture or neurogenic detrusor overactivity was considered a contraindication for surgical treatment of SUI. The degree of male SUI was classified according to the 24-h pad count (grade I: 1–2 pads/day; grade II: 3–5 pads/day; grade III: >5 pads/day). The surgical procedures were performed routinely, as previously described (1, 2). The mean follow-up time of the study was 38 ± 26 months, but was at least 24 months. During the follow-up period, the continence and voiding parameters (24-h pad count, 24-h pad test, uroflowmetry, post-void residual volume), incontinence distress (International Consultation on Incontinence Questionnaire Short Form—ICIQ-SF), device parameters (adjustments), and especially postoperative OAB activity [according to the International Continence Society (ICS), OAB defined as more than 10 micturition's per 24 h (7)] were evaluated. The first follow-up examination was performed in an ambulant setting 4 weeks after the implantation. The implant fill volume was adjusted at intervals of approximately 4 weeks based on the individual requirements of each patient. After achieving the required continence results, further follow-up examinations were carried out every 12 months. The adjustment was performed routinely with application of 1 to 3 ml fill volume until the desired continence result was achieved. Statistical analysis was performed with GraphPad Prism 7® (GraphPad Software, Inc., La Jolla, United States). Data are presented as the mean ± standard deviation (SD) and range. Statistical significance was considered at *p* < 0.05. The bivariate comparisons were conducted with Mann–Whitney *U*-tests (metric variables) or Chi-square tests (categorical variables).

## Results

The total population included 361 patients. An OAB presented 18 patients (4.9%), with one patient already having been diagnosed with a preoperative sensory OAB. The baseline parameters of the total population are shown in Table 1. Regarding the cause of SUI, our total population included patients after radical prostatectomy (82.5%), primary high-intensity focused ultrasound (HIFU) (0.8%), transurethral prostate resection (16.3%) and open adenomectomy (0.3%), as well as one patient who had a previous urethral injury (0.3%). For the group of patients with OAB, the only causes of SUI were radical prostatectomy (12/18, 67.7%) and transurethral prostate resection (6/18, 33.3%). Regarding the degree of urinary incontinence, there were no significant differences between patients with and without OAB. For the total population, 8.9% of the patients presented with a SUI grade I, 59.3% with a SUI grade II and 31.9% with a SUI grade III, whereas in patients with OAB, the distribution was similar: 5.6, 61.1, and 33.3%, respectively. The preoperative parameters are shown in Table 2. For the total population on average, the patients were 69 ± 7.0 (46–94) years old, slightly overweight [body mass index (BMI): 28 ± 3.7 (20–47) kg/m^2^] and clearly presented with comorbidities [Charlson comorbidity index (CCI): 7.2 ± 1.4 (1–12)]. With respect to previous radiotherapy for prostate cancer, previous urinary incontinence surgery, and transurethral resection of a urethral stricture, 25.8, 26, and 23.5% of patients were pre-treated with these measures, respectively. The average number of pads was 5.1 ± 2.9 pads/24 h, with an ICIQ-SF score averaging 16.8 ± 2.0 (12–21), indicating significant urinary incontinence-related distress. In the comparison of the patients with and without OAB, a significant difference was found for the circumstance of previous radiotherapy and previous incontinence surgery, as well as previous transurethral resection of a urethral stricture. Compared to patients without OAB, patients with OAB were more often previously irradiated (38.9 vs. 25.1%, *p* = 0.030), were more often pre-treated for urinary incontinence (61.1 vs. 24.2%, *p* = 0.006) and had more instances of pre-treatment resections for urethral stricture (55.6 vs. 21.9%, *p* = 0.007). There were no significant differences with respect to the other preoperative parameters.

**Table 1:** Baseline characteristics of the patient population (*n* = 361 patients); percentage.

**Category**	**Overall population *n* = 361 pts**	**No OAB *n* = 343 pts**	**OAB *n* = 18 pts**
**Cause of stress urinary incontinence**			
• Radical prostatectomy (%)	298/361 (82.5)	286/343 (83.4)	12/18 (67.7)
• Primary HIFU (%)	3/361 (0.8)	3/343 (0.9)	0
• TUR-P (%)	59/361 (16.3)	53/343 (15.5)	6/18 (33.3)
• Open adenomectomy (%)	1/361 (0.3)	1/343 (0.3)	0
• Urethral injury (%)	1/361 (0.3)	1/343 (0.3)	0
**Degree of stress urinary incontinence**			
• SUI I°: 1–2 pads/day (%)	32/361 (8.9)	31/343 (9)	1/18 (5.6)
• SUI II°: 3–5 pads/day (%)	214/361 (59.3)	203/343 (59.2)	11/18 (61.1)
• SUI III°: >5 pads/day (%)	115/361 (31.9)	109/343 (31.8)	6/18 (33.3)

**Table 2:** Preoperative parameters of the patient population (*n* = 361 patients); mean ± standard deviation (range) or percentage.

**Category**	**Overall population *n* = 361 pts**	**No OAB *n* = 343 pts**	**OAB *n* = 18 pts**	*P*-value^*^ “no OAB” vs. “OAB”
Patient age [years] (range)	69 ± 7.0 (46–94)	69 ± 7 (46–94)	71 ± 6.3 (56–83)	0.146
BMI [kg/m^2^] (range)	28 ± 3.7 (20–47)	27.9 ± 3.6 (20–47)	28.8 ± 4.7 (23–40)	0.137
ASA score (range)	2.2 ± 0.6 (1–4)	2.1 ± 0.6 (1–4)	2.3 ± 0.6 (1–4)	0.145
CCI score (range)	7.2 ± 1.4 (0–12)	7.2 ± 1.3 (1–12)	7.3 ± 1.4 (1–12)	0.178
Primary/secondary radiation (%)	93/361 (25.8)	86/343 (25.1)	7/18 (38.9)	**0.030**
Previous surgery due to SUI (%)	94/361 (26)	83/343 (24.2)	11/18 (61.1)	**0.006**
Previous transurethral resection for urethral stricture (%)	85/361 (23.5)	75/343 (21.9)	10/18 (55.6)	**0.007**
Preoperative 24-h pad count (range)	5.1 ± 2.9 (2–18)	5.1 ± 3.0 (2–18)	4.7 ± 1.9 (2–9)	0.772
Preoperative 24-h pad test [ml] (range)	589 ± 445 (40–2,500)	590 ± 444 (40–2,500)	580 ± 456 (110–2,500)	0.827
Preoperative ICIQ-SF score (range)	16.8 ± 2.0 (12–21)	16.8 ± 2.0 (12–21)	16.9 ± 2.1 (12–21)	0.655

* *Mann–Whitney U–test and Chi-square test (α = 0.05)*.

*Significances highlighted in bold*.

The peri- and postoperative parameters are listed in Table 3. Again initially considered for the total population, ATOMS implantation took on average 51 ± 21 (11–148) minutes and an adjustment was necessary on average 3.4 ± 2.1 (0–9) times. With regard to pad use, the average number decreased from 5.1 ± 2.9 pads preoperatively to 1.3 ± 1.3 pads/day postoperatively. The mean incidence of urinary incontinence distress according to the ICIQ-SF score decreased from 16.8 ± 2.0 to 4.9 ± 4.5 (0–20) after surgery. Because ATOMS is a system that achieves continence by suburethral compression, it produces an average uroflowmetry of 14.7 ± 3.9 (4–35) ml/s and a mean postoperative post-void residual volume of 8 ± 23 (0–200) ml.

**Table 3:** Peri- and postoperative parameters of the patient population (*n* = 361 patients); mean ± standard deviation (range).

**Category**	**Overall population *n* = 361 pts**	**No OAB *n* = 343 pts**	**OAB *n* = 18 pts**	P-value^*^ “no OAB” vs. “OAB”
Operative time [min] (range)	51 ± 21 (11–148)	51 ± 21 (11–148)	48 ± 21 (21–138)	0.364
Postoperative adjustments (range)	3.4 ± 2.1 (0–9)	3.5 ± 2.1 (0–9)	2.5 ± 1.8 (0–7)	**0.067**
Postoperative cushion volume [ml] (range)	14.4 ± 6.4 (5–25)	14.5 ± 6.5 (5–25)	13.4 ± 5.6 (6–25)	0.263
Postoperative 24-h pad count (range)	1.3 ± 1.3 (0–8)	1.2 ± 1.4 (0–8)	1.4 ± 1.3 (0–5)	0.446
Postoperative 24-h pad test [ml] (range)	70 ± 147 (0–1,500)	68 ± 142 (0–1,500)	91 ± 192 (0–550)	0.278
Postoperative uroflowmetry [ml/s] (range)	14.7 ± 3.9 (4–35)	14.6 ± 3.8 (4–35)	15.5 ± 4.3 (10–30)	0.490
Postoperative residual volume [ml] (range)	8 ± 23 (0–200)	8 ± 24 (0–200)	6 ± 10 (0–40)	0.969
Postoperative ICIQ-SF score (range)	4.9 ± 4.5 (0–20)	5 ± 4.5 (0–20)	3.6 ± 4 (0–16)	**0.105**

* *Mann–Whitney U-test (α = 0.05)*.

*Tendencies highlighted in bold*.

Comparing the patient groups with and without OAB, no significant differences were found for the peri- or postoperative parameters. For adjustment of the ATOMS device as well as the postoperative ICIQ-SF score, a statistically tendency was observed. In contrast to patients without OAB, patients with OAB were adjusted 2.5 ± 1.8 times vs. 3.5 ± 2.1 times (*p* = 0.067) and had a slightly lower urinary incontinence-related burden of suffering at 3.6 ± 4 vs. 5 ± 4.5 (*p* = 0.105), despite the symptoms of OAB. The success rates of the ATOMS device are shown in Table 4. In the total population, implantation of the ATOMS urinary incontinence system resulted in 54% of patients becoming “dry” (0 or one “safety pad”/day), while 28% of patients achieved at least an “improvement” of more than 50% (1–2 pads/day), which corresponds to a total success rate of 82%. For the patients of the OAB group, this outcome was similar, with 50% “dryness” and 27.8% “improvement,” corresponding to a total success rate of 77.8%.

**Table 4:** Postoperative degree of stress urinary incontinence of the patient population (*n* = 361 patients); percentage.

**Category**	**Overall population *n* = 361 pts**	**No OAB *n* = 343 pts**	**OAB *n* = 18 pts**
• Dry: 0-“safety pad”/day	195/361 (54)	186/343 (54.2)	9/18 (50)
• Improvement: 1–2 pads/day (>50% improvement compared to baseline)	101/361 (28)	96/343 (28)	5/18 (27.8)
• Failure: >2 pads/day (<50% improvement compared to baseline)	65/361 (18)	61/343 (17.8)	4/18 (22.2)

**Figure 1:**
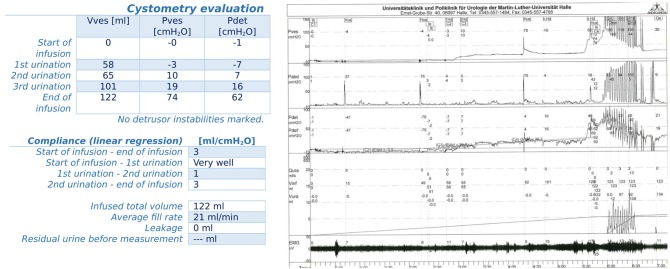
Urodynamic evaluation of a patient with a newly diagnosed, derailed and primarily insulin-dependent type II diabetes mellitus and OAB voiding dysfunction; urodynamic evaluation showed a low-compliance urinary bladder.

The patient with preoperative sensory OAB (condition after TUR-P and subsequent curative radiotherapy for an incidental prostate cancer; had a daily micturition frequency and nocturia at the time of first presentation before urinary incontinence surgery of about <11 and <3 times, respectively) was already preoperatively medicated with antimuscarinic (trospium chloride), leading to significant relief of OAB symptoms (daily micturition frequency maximum of 7x, nocturia maximum of 1x), which ultimately made incontinence surgery possible. For patients with newly postoperative OAB, a further evaluation was carried out by means of a new urodynamic evaluation, whereby in 16 patients a sensory OAB and in another patients a low-compliance urinary bladder (Figure 1) in a newly diagnosed, derailed, and primarily insulin-dependent type II diabetes mellitus were found (blood glucose in new diabetic diagnoses: >30 mmol/l). In the 16 patients with sensory OAB, antimuscarinic drugs were given in standard doses, which improved the OAB symptoms in all 16 patients. However, in the case of the patient with the low-compliance bladder, the administration of antimuscarinic drugs was frustrating, so transurethral botulinum toxin type A (100 units) was injected. No post-interventional readjustment of the ATOMS fill volume had to be made (continuing 25 ml). The patient was significantly improved after botulinum toxin injection in terms of OAB, had no post-void residual volume and required one to a maximum of 3 pads per day, and the injection has been repeated 3 times to date.

In the identification of possible risk factors for OAB after ATOMS implantation, a positive correlation was found only for a previous radiotherapy or previous urinary incontinence surgery, as well as resection of a urethral stricture. The present data does not suggest any evidence that the ATOMS device itself contributes to OAB.

## Discussion

The surgical treatment of male SUI is highly interesting but also challenging in functional urology. On the one hand, the patients are mostly severely impaired (4, 8, 9), on the other hand, different medical devices in addition to the artificial sphincter have been developed. The effectiveness of these devices for male SUI is dependent on the degree of incontinence and the condition of the patient. These devices include AdVance and AdVanceXP (Boston Scientific, USA), ProACT (Uromedica, USA), Argus Classic and ArgusT (Promedon, Argentina), MRS Remeex (Neomedic, Spain) and ATOMS (A.M.I., Austria), among others (10). The goal of all urinary incontinence devices is to support or even replace the external sphincter function. For adjustable urinary incontinence systems, a technical distinction is made between the retropubic and transobturator approach and bladder neck balloon placement. The mechanism of action of the adjustable urinary incontinence systems is similar and involves a permanent compression of the urethra or bladder neck, in the sense of supporting the external residual sphincter function and therefore supporting basic continence. Subsequently, it is only a static support of the sphincter function, with the charm that this compression can be “readjusted” if necessary. To what extent symptoms of a “*de novo*” OAB can arise as a result, or consequently, which risk factors lead to OAB after ATOMS implantation, are unknown to date. For female urinary incontinence surgery, we know the phenomenon of postoperative “*de novo*” urgency (11–13), which is generated by the introduction of foreign material in the anterior vaginal wall, paraurethral or close to the bladder neck, and which based on the literature, occurs with a probability of 9–33% (13–16). The causes of this *de novo* urgency are in the simplest case a urinary tract infection, but usually a foreign body irritation or even a malposition with possibly consecutive perforation of the urethra or bladder neck by the foreign material (12, 17). There are no differences between the retropubic and transobturator approaches of female mid-urethral slings (18). For male urinary incontinence surgery, there has been little research to date on symptoms of *de novo* urgency (19–22) and especially not for the suburethrally adjustable urinary incontinence system ATOMS. The present study therefore examined the prevalence, status and therapy of OAB after the urinary incontinence system ATOMS.

Our total population included 361 patients. An OAB presented 18 patients (4.9%), with one patient already diagnosed with preoperative sensory OAB. In the bivariate analysis of all parameters, there was a positive correlation with a *de novo* urgency for previous radiotherapy (*p* = 0.030) and urinary incontinence surgery (*p* = 0.006) as well as for resection of a urethral stricture (*p* = 0.007). The influence of radiation on the urinary bladder can generally be considerable, although the pathophysiology of so-called “radiation cystitis” is still poorly understood. Several damage-inducing mechanisms have been described. Thus, ionizing radiation traumatizes the urothelium, the smooth detrusor muscle cells and the vascular supply to the urinary bladder (23, 24). Clinically, in addition to acute inflammation, ionizing radiation damage leads to a decrease in urinary bladder compliance and bladder capacity. Due to its low cell turnover, the bladder is very sensitive to ionizing radiation (25). Clinically, this leads to the “classic” symptoms of cystitis with dysuria, pollakiuria, and “urgency,” but with sterile urine. Late bladder reactions include decreased bladder compliance and bladder capacity, as well as the development of malignancies (26, 27). In our study, OAB patients were pre-irradiated compared to patients without OAB in 38.9 vs. 25.1% of patients (*p* = 0.030). However, the primary genesis of urinary incontinence can also have a significant influence on the development of urgency symptoms. Investigations on “voiding dysfunctions” after transurethral resection (28) as well as after radical prostatectomy (29) have shown that up to 80% of the patients affected by these postoperative micturition symptoms show urodynamic abnormalities, especially in sense of detrusor overactivity, and somewhat less in sense of detrusor hypocontractility (30). In our study, the group of patients with OAB included only those after radical prostatectomy (12/18, 67.7%) or after transurethral prostate resection (6/18, 33.3%). However, patients with other underlying causes of urinary incontinence were relatively under-represented in our study. In only 4 of 361 patients (1.1%), SUI resulted from primary HIFU, open adenomectomy or urethral trauma. However, it is possible that the cause of SUI has influenced urgency symptoms. The prevalence of OAB after previous urinary incontinence surgery was also statistically significant, whereby patients with OAB were pre-treated for urinary incontinence in 61.1 vs. 24.2% of patients (*p* = 0.006). In the literature, there is no evidence in this regard, so the present data show a possible connection for the first time. There is also no evidence to date regarding the influence of previous urethral stricture resection on the development of urgency symptoms. A detailed look at the data shows, however, that patients with prior radiotherapy needed resection of urethral stricture in one third (28/93, 30.1%), so urethral scar formation is very likely to have an indirect connection after previous irradiation.

The prevalence of postoperative OAB in other urinary incontinence systems for the treatment of male SUI can be found with AdVance, AdVanceXP, InVance (19, 20) and artificial sphincter (21). Thus, Collado et al. (19) found in their retrospective study (study period: 02/08-10/14) a postoperative *de novo* urgency in 22 patients (16%) out of 24 who received an AdVance and 70 who received an AdVanceXP device. In a subsequent study, comparing AdVance and InVance implantation (20), they also found a “*de novo*” OAB for the InVance (31 implants) in 22.5% of cases (7 patients). For the artificial sphincter, Serag et al. (21) and Ko et al. (22) observed a postoperative OAB in 6 and 37.5% of their patients, respectively. However, in the further literature on artificial urethral sphincter, as well as for other adjustable urinary incontinence systems, there is no information on postoperative *de novo* urgency symptoms (31–33). In addition, for the urinary incontinence system ATOMS in particular, only evidence on the risk factors for a therapeutic failure and information on patient satisfaction can be found. Friedl et al. (3) found that patients with primary ATOMS implantation and without previous radiotherapy have a better therapeutic outcome, while Angulo et al. (34) showed that patient satisfaction correlates positively with postoperative dryness (*p* < 0.0001), lower urinary incontinence (*p* = 0.007), less postoperative pain (*p* = 0.0018) and a lack of complications (*p* = 0.007).

Our data further implicate a potential impact on the adjustment of an ATOMS device. For example, patients with OAB tended to be adjusted less often than patients without OAB [2.5 ± 1.8 vs. 3.5 ± 2.1 times (*p* = 0.067)]. Upon further evaluation, the mean uroflow [patients without OAB: 14.6 ± 3.8 (4–35) ml/s vs. patients with OAB: 15.5 ± 4.3 (10–30) ml/s], residual post-void volume [patients without OAB: 8 ± 24 (0–200) ml vs. patients with OAB: 6 ± 10 (0–40) ml] and pad count [patients without OAB: 1.2 ± 1.4 (0–8) pads/day vs. patients with OAB: 1.4 ± 1.3 (0–5) pads/day] were similar for both groups (*p* > 0.05). Equally striking, patients with OAB had a lower urinary incontinence-related burden of suffering according to ICIQ-SF [3.6 ± 4 vs. 5 ± 4.5 (*p* = 0.105)], despite the OAB symptoms and despite the similar number of pads/day. As mentioned by Michel et al. (35), it can be assumed that OAB and urinary incontinence do not necessarily correlate with each other.

In summary, a postoperative OAB after ATOMS device implantation can occur, but its prevalence is low. According to the available data, the cause of this “*de novo*” OAB is not the urinary device itself but rather the origin of the urinary incontinence, as well as previously prescribed therapy. For preoperative diagnostics prior to ATOMS implantation, patients with risk factors should therefore be aware of any pre-existing OAB, and this information should be included in the concealing process prior to surgery. From the authors' point of view, it is therefore advisable that a bladder capacity of at least 150 ml or, even better, 250 ml should be available to avoid postoperative OAB. Ko et al. (22) even recommend 300 ml of bladder capacity. However, one must add that there is no further evidence regarding this necessary bladder capacity (10). A preoperative urodynamic assessment including calculation of the maximum bladder capacity seems to be recommended in any case. However, OAB after ATOMS device implantation is usually well-treated and does not have any major therapeutic consequences for the affected patient.

The limitations of this study are the lack of a control group as well as the heterogeneous patient population, which ultimately corresponds to the daily practice, but which may have influenced both, the patient outcome and the prevalence of postoperative urgency symptoms. Of course, a critical consideration of your own preoperative diagnosis and results applies. Therefore, it is possible in principle that pre-existing OAB in the preoperative diagnosis was not accurately detected. Further and larger investigations must follow.

## Conclusion for Clinical Practice

The implantation of an adjustable transobturator urinary incontinence system (ATOMS) is an established surgical procedure for the treatment of persistent male SUI. A postoperative “*de novo*” OAB, in the sense of a sensory OAB, can occur, but is rare and has no closer correlation to the urinary device, but rather “*de novo*” OAB may be due to the urinary incontinence-related underlying disease and the pre-treatments that an individual patient received. In this regard, previous radiation has a particularly significant impact.

## Data Availability Statement

All datasets generated for this study are included in the article/supplementary material.

## Ethics Statement

This study was carried out in accordance with the recommendations of the ethics commission of the Martin-Luther University Halle-Wittenberg, with written informed consent from all patients. All patients gave written informed consent in accordance with the Declaration of Helsinki. The protocol was approved by the ethics commission of the Martin-Luther University Halle-Wittenberg.

## Author's Note

SS was previously listed as Sandra Mühlstädt in PubMed.

## Author Contributions

SS: protocol development, data collection and analysis, and manuscript writing. WB: data collection and analysis, and manuscript writing. NM and CB: manuscript editing. PF: protocol development and manuscript editing.

### Conflict of Interest

SS: Scientific lecturing of the surgical incontinence workshop “Treatment of male incontinence using the adjustable transobturator A.M.I. ATOMS system” at the Department of Urology and Kidney Transplantation, Martin Luther University, Halle (Saale), Germany, and secondary activity as a “Flying-Doc” for the manufacturer. WB: Inventor and financial contract with the manufacturer. The remaining authors declare that the research was conducted in the absence of any commercial or financial relationships that could be construed as a potential conflict of interest.
